# Chemoreceptors with C-terminal pentapeptides for CheR and CheB binding are abundant in bacteria that maintain host interactions

**DOI:** 10.1016/j.csbj.2020.07.006

**Published:** 2020-07-16

**Authors:** Álvaro Ortega, Tino Krell

**Affiliations:** a Department of Biochemistry and Molecular Biology ‘B’ and Immunology, Faculty of Chemistry, University of Murcia, Regional Campus of International Excellence “Campus Mare Nostrum”, Murcia, Spain; b Department of Environmental Protection, Estación Experimental del Zaidín, Consejo Superior de Investigaciones Científicas, Granada, Spain

**Keywords:** Chemotaxis, Chemoreceptor, CheR, Chemosensory pathway, C-terminal pentapeptide

## Abstract

Chemosensory pathways represent a major prokaryotic signal transduction mechanism that is based on signal sensing by chemoreceptors. An essential feature of chemosensory pathways is the CheR and CheB mediated control of chemoreceptor methylation causing pathway adaptation. At their C-terminal extension the Tar and Tsr model chemoreceptors contain a pentapeptide that acts as an additional CheR and CheB binding site. The relevance of this pentapeptide is poorly understood since pentapeptide removal from Tar/Tsr causes receptor inactivation, whereas many other chemoreceptors do not require this pentapeptide for correct function. We report here a bioinformatic analysis of pentapeptide containing chemoreceptors. These receptors were detected in 11 bacterial phyla and represent approximately 10% of all chemoreceptors. Pentapeptide containing chemoreceptors are mainly found in Gram-negative bacteria, are of low abundance in Gram-positive species and almost absent from archaea. Almost 50% of TarH (Tar homologue) ligand binding domain containing chemoreceptors possess pentapeptides, whereas chemoreceptor families with other ligand binding domains are devoid of pentapeptides. The abundance of chemoreceptors with C-terminal pentapeptides correlated negatively with the number of chemoreceptor genes per genome. The consensus sequence reveals a negative net charge for many pentapeptides. Pentapeptide containing chemoreceptors are very abundant in the order Enterobacterales, particularly in the families Pectobacterium and Dickeya, where they represent about 50% of the total number. In contrast, bacteria with primarily free living lifestyles have a reduced number of pentapeptides, such as approximately 1% for Pseudomonadales. It is proposed that pentapeptide function is related to mechanisms that permit host interaction.

## Graphical abstract

**Figure:**
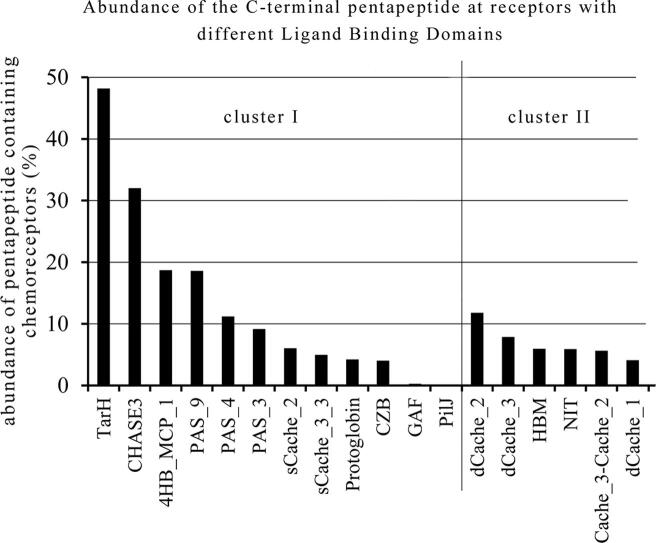

## 1 Introduction

**Fig. 1:**
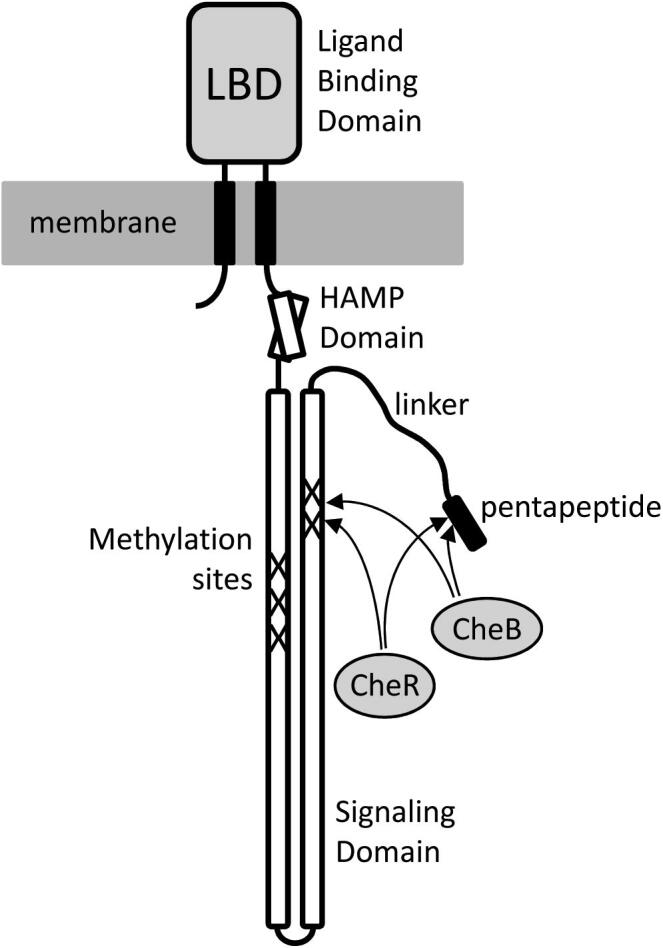
Schematic representation of a chemosensory pathway. HAMP: domain found in histidine kinases, adenylyl cyclases, methylaccepting proteins and phosphatases.

Chemosensory pathways are among the most abundant prokaryotic signal transduction mechanisms [1], [2], [3]. Typically, chemoreceptors recognize signal molecules at their ligand binding domain (LBD)^1^ that lead to a modulation of the activity of the CheA autokinase and subsequently alters the transphosphorylation to the CheY response regulator (Fig. 1). Pathway output is defined by the ratio of phosphorylated to unphosphorylated CheY [4]. Most chemosensory pathways appear to mediate chemotaxis whereas other pathways have been associated with type IV pili-based motility or were shown to exert alternative cellular functions such as the control of second messenger levels [5], [6]. More than half of the bacterial genomes analysed were found to contain genes for chemosensory signaling [2]. Chemosensory signaling mechanisms can be very complex which is reflected in a frequently elevated number of chemoreceptors, up to 88, and the existence of multiple parallel pathways, up to 8, with different functions [3], [7].

An integral part of chemosensory pathways is their capacity to adapt their sensitivity to the present signal concentration. The predominant adaptation mechanism is based on the methylation and demethylation of several glutamyl residues at the chemoreceptor signaling domain, catalysed by the CheR methyltransferase and CheB methylesterase, respectively [4]. The importance of chemoreceptor methylation is illustrated by the fact that both enzymes are among the core proteins of chemosensory pathways, i.e. present in almost all pathways [2]. The *E. coli* chemoreceptors contain four or five methylation sites that are located approximately in the middle of the long rod formed by the signaling domain (Fig. 1). Data indicate that CheR binds with a relatively low affinity (*K*_D_ in the range of hundreds of µM) to these methylation sites [8], [9].

Of the *E. coli* chemoreceptors, the high-abundant Tar and Tsr, possess a C-terminal pentapeptide, NWETF, that is tethered to the C-terminal end of the chemoreceptor signaling domain via an unstructured linker of approximately 35 amino acids [10]. CheR was found to bind to this pentapeptide with an affinity of approximately 2 µM [8], [11], an affinity that is significantly higher than that for the methylation site. It was proposed that CheR binding to the pentapeptide enhanced the local CheR concentration leading to optimal adaptation [12], [13]. Mutation or removal of this pentapeptide from Tar and Tsr largely reduced methylation *in vivo* and *in vitro* and abolished chemotaxis [12], [14], [15], [16]. The structure of CheR in complex with the pentapeptide has been solved. The structure reveals the central importance of aromatic amino acids at positions 2 and 5 of this peptide for binding [17], a notion that is also supported by site-directed mutagenesis experiments [18].

In contrast to CheR, CheB binds to this pentapeptide with much lower affinity (*K*_D_ = 160 µM) [19]. However, removal of this pentapeptide had also a detrimental effect on the CheB mediated activities, demethylation and deamidation, *in vivo* and *in vitro* [16], [20]. In contrast to CheR, the affinity of CheB for the pentapeptide is too low as to increase the local concentration, but the CheB-pentapeptide interaction was found to stimulate methylesterase activity [19]. While the pentapeptide is essential for Tar and Tsr function, many other chemoreceptors have been identified that lack C-terminal pentapeptides and that mediate strong chemotactic responses [21], [22], [23], [24], [25]. The paradoxical situation that the pentapeptide is essential for some receptors while dispensable for others is not well understood and has motivated the present study.

What is known about pentapeptide containing chemoreceptors in other species? *Pseudomonas aeruginosa* has 26 chemoreceptors that feed into four chemosensory pathways [26]. McpB/Aer2 is the sole chemoreceptor that contains a C-terminal pentapeptide [27] and is predicted to be the sole chemoreceptor that feeds into the che_2_ pathway [26]. Of the four CheR paralogues, CheR_2_, the methyltransferase of the che_2_ pathway, was the only paralogue that bound the pentapeptide [27]. It has therefore been concluded that the pentapeptide-CheR interaction is a mechanism that permits the targeting of a specific chemoreceptor with a specific CheR [27]. The pentapeptide was shown to bind to the β-subdomain of CheR and several studies have identified features in this subdomain that permit to distinguish between pentapeptide dependent and pentapeptide independent CheRs [9], [27], [28]. In *E. coli*, pentapeptides are only present at the high abundance receptors Tar and Tsr, but not in Tap and Trg that are of lower abundance [29]. Of the eight chemoreceptors of *Sinorhizobium meliloti,* four contain a pentapeptide. However, in this species no correlation was observed between the cellular abundance of chemoreceptors and the presence of the pentapeptide [30].

An analysis based on 167 genomes from the year 2007 indicated that chemoreceptors with the C-terminal pentapeptide are found in α, β, γ and δ Proteobacteria and Spirochetes [9]. Considering the number of genomes sequenced since then, we present here an analysis of the totality of currently available pentapeptide containing chemoreceptors. We determine the phylogenetic distribution of these chemoreceptors and investigate relationships between their abundance and receptor topology, genome chemoreceptor gene content, type of sensor domain and bacterial lifestyle. A pentapeptide consensus sequence is presented that reveals interesting features. Data presented here provide novel insight into the relevance of pentapeptides in chemoreceptor function.

## 2 Results and discussion

### 2.1 Approximately 10% of bacterial chemoreceptors contain a C-terminal pentapeptide

In an initial step we retrieved all available chemoreceptor sequences. Analysis of all protein sequences deposited in the TrEMBL database resulted in the detection of 247,387 chemoreceptor sequences. 237,805 chemoreceptors were identified in 17,322 bacterial proteomes, corresponding to a mean of 13.7 ± 13.0 chemoreceptors per proteome (Supp. Table 1). 3896 chemoreceptors were detected in 595 archaeal proteomes, corresponding to a mean of 6.54 ± 5.86 receptors per proteome (Supp. Table 2). The remaining chemoreceptors were mostly from unclassified species and a few eukaryotic sequences that were identified as false positive hits. The determined abundance of chemoreceptors in bacteria and archaea is similar to that of a previous study [31].

Terminal pentapeptides were detected in 25,635 bacterial chemoreceptors corresponding to 10.78% of the total number of bacterial chemoreceptors (Supp. Table 1). This number is close to the estimation of 10% from the analysis of 167 genomes [9]. Only four archaeal chemoreceptors contained a pentapeptide (Supp. Table 2). BLAST searches revealed that these are no bacterial contaminations and information shown in Supp. Fig. 1 indicates that three of these four sequences may indeed correspond to archaeal chemoreceptors with pentapeptides.

## 3 Transmembrane and cytosolic chemoreceptors possess C-terminal pentapeptides

Chemoreceptors differ in their topology and the cellular compartment in which they sense the signals [31], [32]. The prototypal transmembrane receptor contains two transmembrane helices that flank the extracytosolic LBD, whereas the family of cytosolic chemoreceptors lacks transmembrane regions and senses stimuli in the cytosol [32]. Of the chemoreceptors retrieved, 23.3% were cytosolic and the rest of transmembrane nature, which is an estimate similar to a previous report [32]. Approximately 7.5% of cytosolic chemoreceptors possess a C-terminal pentapeptide whereas this ratio was with 11.5% slightly higher for transmembrane receptors, indicating that chemoreceptors that sense signals in the cytosol and the extracytoplasmic space possess C-terminal pentapeptides.

## 4 The abundance of chemoreceptors with C-terminal pentapeptides correlates negatively with the number of chemoreceptors per genome

**Fig. 2:**
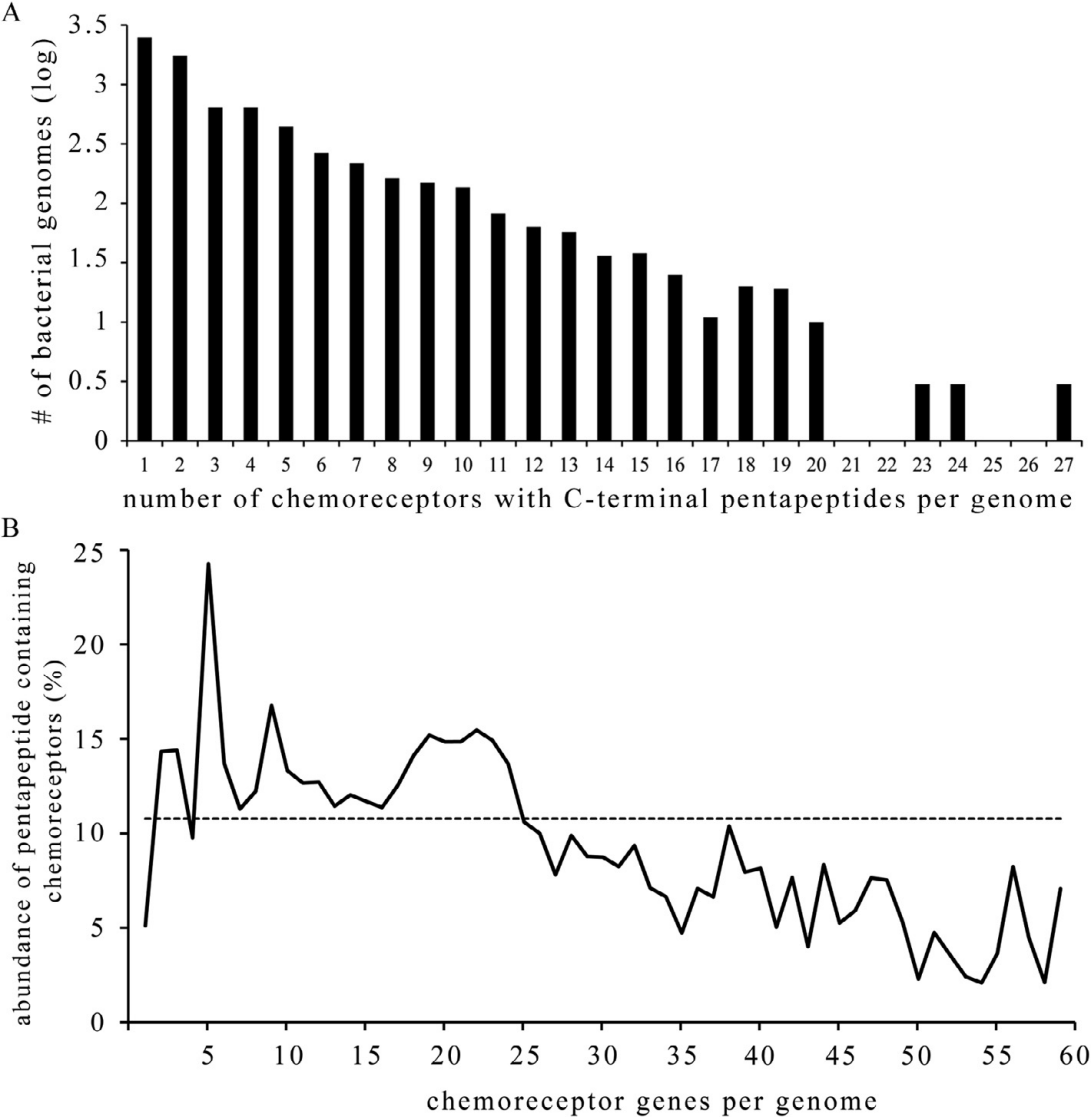
Abundance of C-terminal pentapeptide containing chemoreceptors per genome. A) Abundance of bacterial genomes with different total numbers of pentapeptide containing chemoreceptors per genome. B) Abundance of C-terminal pentapeptide containing chemoreceptors per genome with respect to the total number of chemoreceptors as function of the total number of chemoreceptor genes per genome. The horizontal line marks the bacterial average of 10.78%.

Bacterial and archaeal genomes differ largely in the number of chemoreceptor genes. Whereas some possess a single chemoreceptor gene, others harbour more than 80 [3], [33], [34]. Fig. 2A shows the absolute number of pentapeptide containing chemoreceptor per genome. Most abundant are genomes that contain a single chemoreceptor with pentapeptide. There is a gradual decrease in the number of genomes as the total number of pentapeptide containing chemoreceptors per genome increases until genomes that contain 20 such receptors. This appears to be an upper limit since very few genomes have more than 20 such chemoreceptors. Supp. Table 1 shows that 99.5% of bacterial genomes possess less than 60 chemoreceptor genes. The abundance of pentapeptide containing chemoreceptors as a function of the total number of chemoreceptor genes per genome is illustrated in Fig. 2B. Data show that there is a negative correlation between the abundance of pentapeptide containing chemoreceptors and the number of chemoreceptor genes per genome. The Karl Pearson correlation of data shown in Fig. 2B was of −0.77, indicative of a high negative correlation.

## 5 Almost half of TarH domain containing chemoreceptors possess a C-terminal pentapeptide

**Fig. 3:**
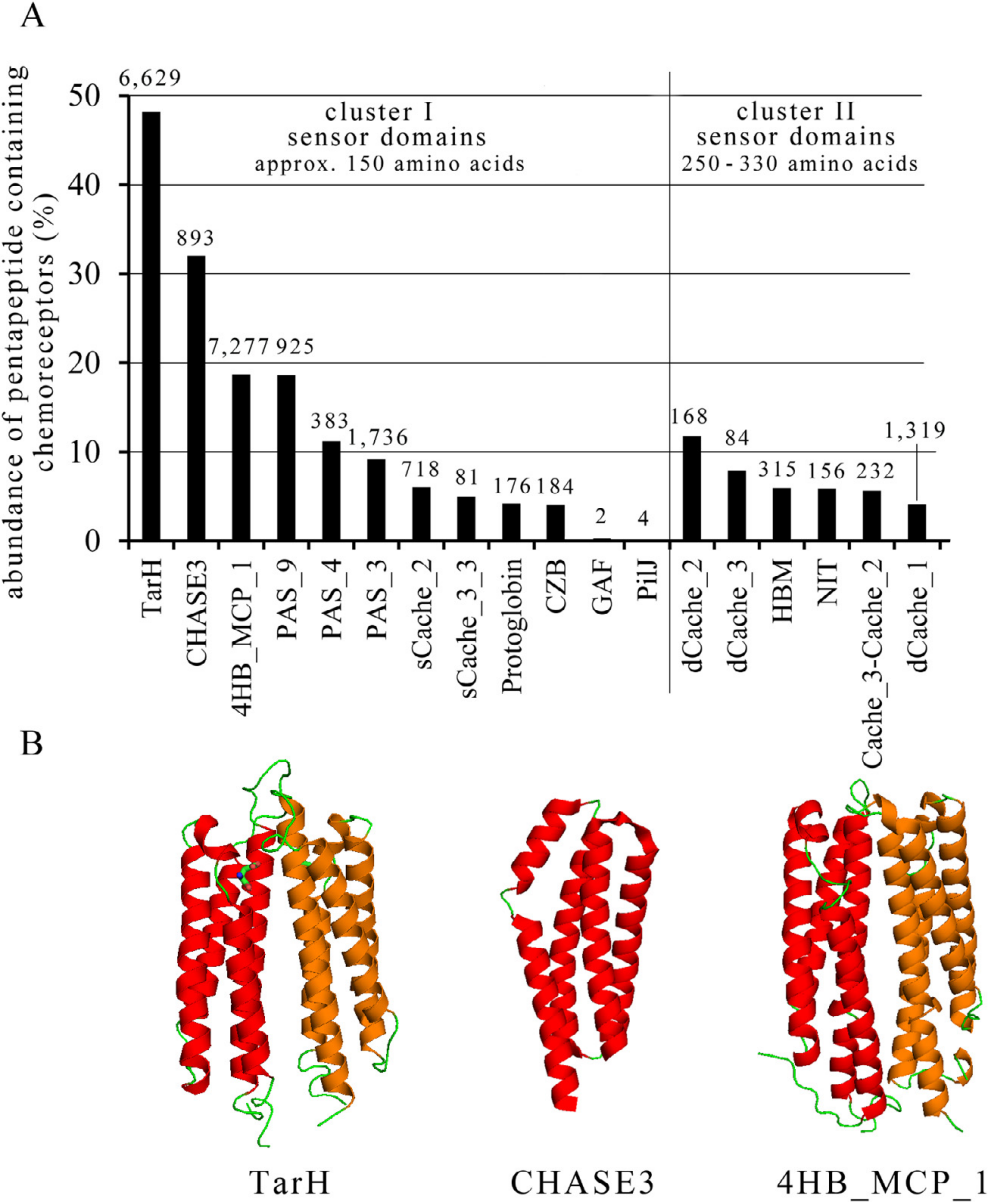
Abundance of C-terminal pentapeptide containing chemoreceptors with respect to the total number of chemoreceptors as function of the LBD type (A). The total number of C-terminal pentapeptide containing chemoreceptors is shown above the columns. Domains shown correspond to the most abundant LBDs in chemoreceptors [35] B) Representative members of the LBD families with highest abundance of pentapeptides. Shown are 3D structures of the LBDs of the Tar chemoreceptor (TarH, pdb ID: 1VLT), the HK9 histidine kinase (CHASE3, pdb ID 3VA9) and a homology model of the LBD of the PA1251 chemoreceptor of *P. aeruginosa* (4HB_MCP_1), generated using Swiss-Model [48].

Chemoreceptors employ at least 80 different types of LBD that can be classified into cluster I (approx. 150 amino acids) and cluster II (approx. 250–330 amino acids) [31], [35]. We have classified chemoreceptors according to the type of their LBD and the abundance of pentapeptide containing chemoreceptors for the most populated families is shown in Fig. 3. Interestingly, almost 50% of TarH domain containing chemoreceptors possess a C-terminal pentapeptide (Fig. 3A). This domain family comprises Tar LBD homologs and the Tar chemoreceptor is the primary model protein to study chemoreceptors [4]. Other families that contain a high percentage of pentapeptides are those containing a CHASE3 and 4HB_MCP_1 type LBD. Interestingly, these three LBD families all form four helix bundle structures (Fig. 3B) indicating that chemoreceptors that contain this fold possess significantly more C-terminal pentapeptides than the bacterial average. In contrast, other families like chemoreceptors containing GAF or PilJ LBDs contain an insignificant number of C-terminal pentapeptides. The largest superfamily of extracellular sensor domains in prokaryotes is formed by Cache domains that can be classified into the families of single-module sCACHE and double-module dCache domains [36]. The abundance of pentapeptides in this superfamily is well below the bacterial average (Fig. 3A). There is a significant amount of functional, biochemical and structural information available on the very populated family of dCACHE_1 containing chemoreceptors [21], [37], [38], [39]. However, only approximately 4% of dCACHE_1 containing chemoreceptors possess C-terminal pentapeptides (Fig. 3A).

## 6 Pentapeptides are always fused to the C-terminus of an MCP, regardless whether the last domain is an LBD or signaling domain

**Fig. 4:**
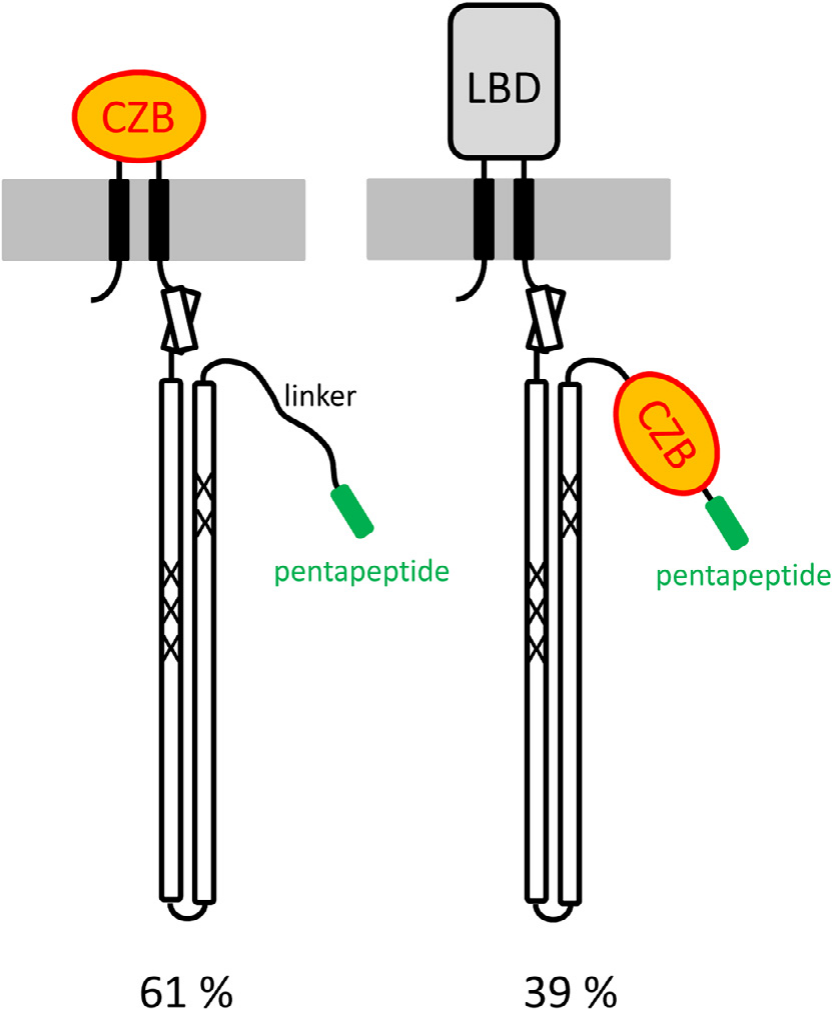
Schematic representation of chemoreceptors that contain a pentapeptide fused to the signaling domain or the CZB zinc binding domain. The individual proteins are listed in Supp. Table 3.

The prototypal chemoreceptor topology comprises a LBD at the N-terminal part and the so far characterized pentapeptide containing chemoreceptors possess a pentapeptide that is fused to the C-terminal extension of the signaling domain. However, there is a poorly characterized chemoreceptor subfamily that contains an LBD at the C-terminal extension. Inspection of the Pfam database has shown that there are at least 17 different types of LBD that are fused to the C-terminal part of the chemoreceptor (Supp. Fig. 2). The question is thus whether the C-terminal pentapeptide is always fused to the C-terminus of an MCP, regardless whether the last domain is an LBD or signaling domain. To address this issue we have analysed chemoreceptors that contain a zinc binding CZB domain [40] that corresponds by far to the most populated chemoreceptor family with a C-terminal LBD (Supp. Fig. 2). In total we detected 184 chemoreceptors that contain a CZB domain and a C-terminal pentapeptide (Supp. Table 3). In 61% of these receptors the CZB domain was at the N-terminal part of the protein and the pentapeptide was fused to the signaling domain (Fig. 4, Supp. Table 3). However, in the remaining 39% the CZB domain was C-terminal to the signaling domain and the pentapeptide was fused to the C-terminal extension of the CZB domain (Fig. 4). Therefore, the pentapeptide can either be fused to the signaling domain or to sensor domains that follow in sequence the signaling domain.

## 7 Bacteria that inhabit the human intestinal flora possess a high abundance of pentapeptide containing chemoreceptors

**Fig. 5:**
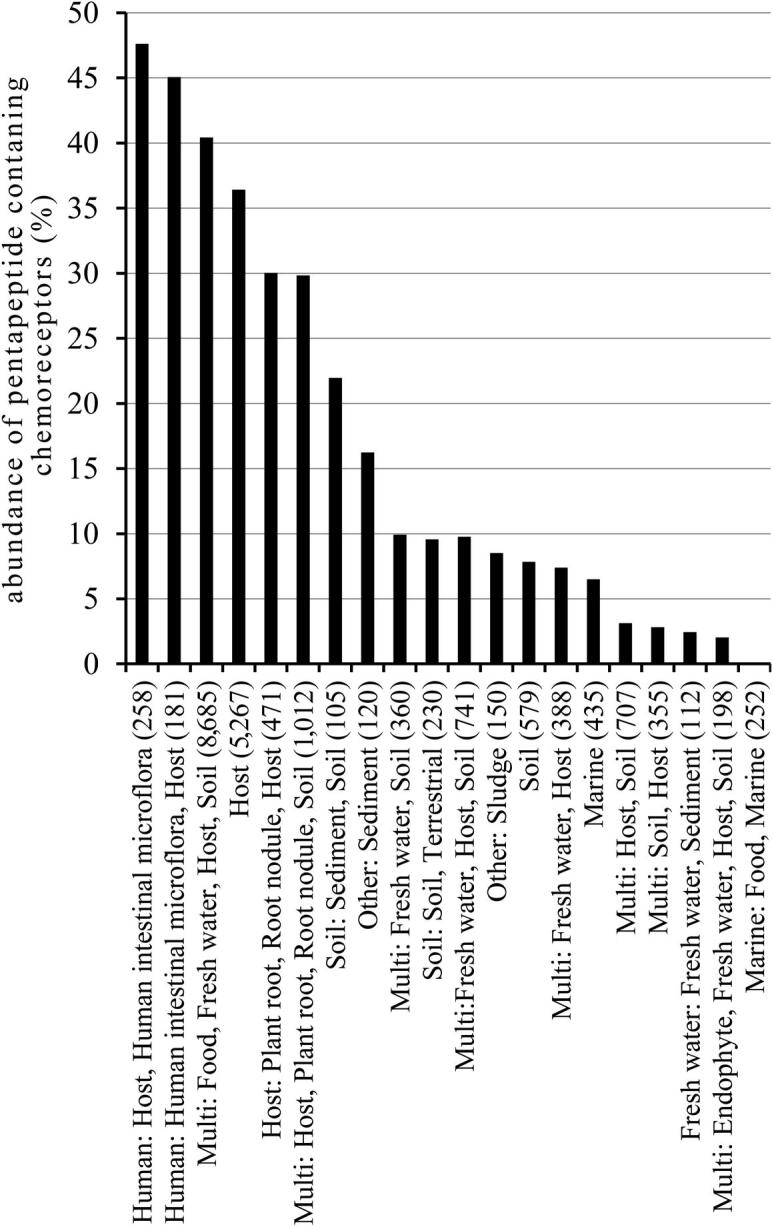
Abundance of pentapeptide containing chemoreceptors with respect to the total number of chemoreceptors as a function of the bacterial habitat. Chemoreceptor containing strains were classified according to their preferred ecological habitat using fusionDB [41]. Shown are the most populated categories. The number of chemoreceptors per category is indicated in brackets.

Chemoreceptor containing bacterial strains were submitted to an analysis by the fusionDB database [41] permitting a classification according to the preferred habitat. This analysis is not always unambiguous since many bacterial species are present in multiple environments. The abundance of pentapeptide containing chemoreceptors in the most populated categories is shown in Fig. 5. It appears that the six most populated categories contain the term “host” in the description and the two most abundant categories were defined as “human intestinal microflora”. In these two categories the abundance of pentapeptide containing chemoreceptors was superior to 40%. However, we also found that TarH domain containing chemoreceptors are very abundant in strains of the order of Enterobacterales where they represent 47% of the total, as compared to the bacterial average of about 5%. Since pentapeptides are abundant in TarH domain containing receptors (Fig. 3), it remains to be established whether the elevated presence of pentapeptides in bacteria inhabiting the human intestine is related to a particular lifestyle or the abundance of the chemoreceptor family with a TarH sensor domain in Enterobacterales, or both.

## 8 Almost a quarter of all pentapeptide containing chemoreceptors is found in strains of the order Enterobacterales

**Fig. 6:**
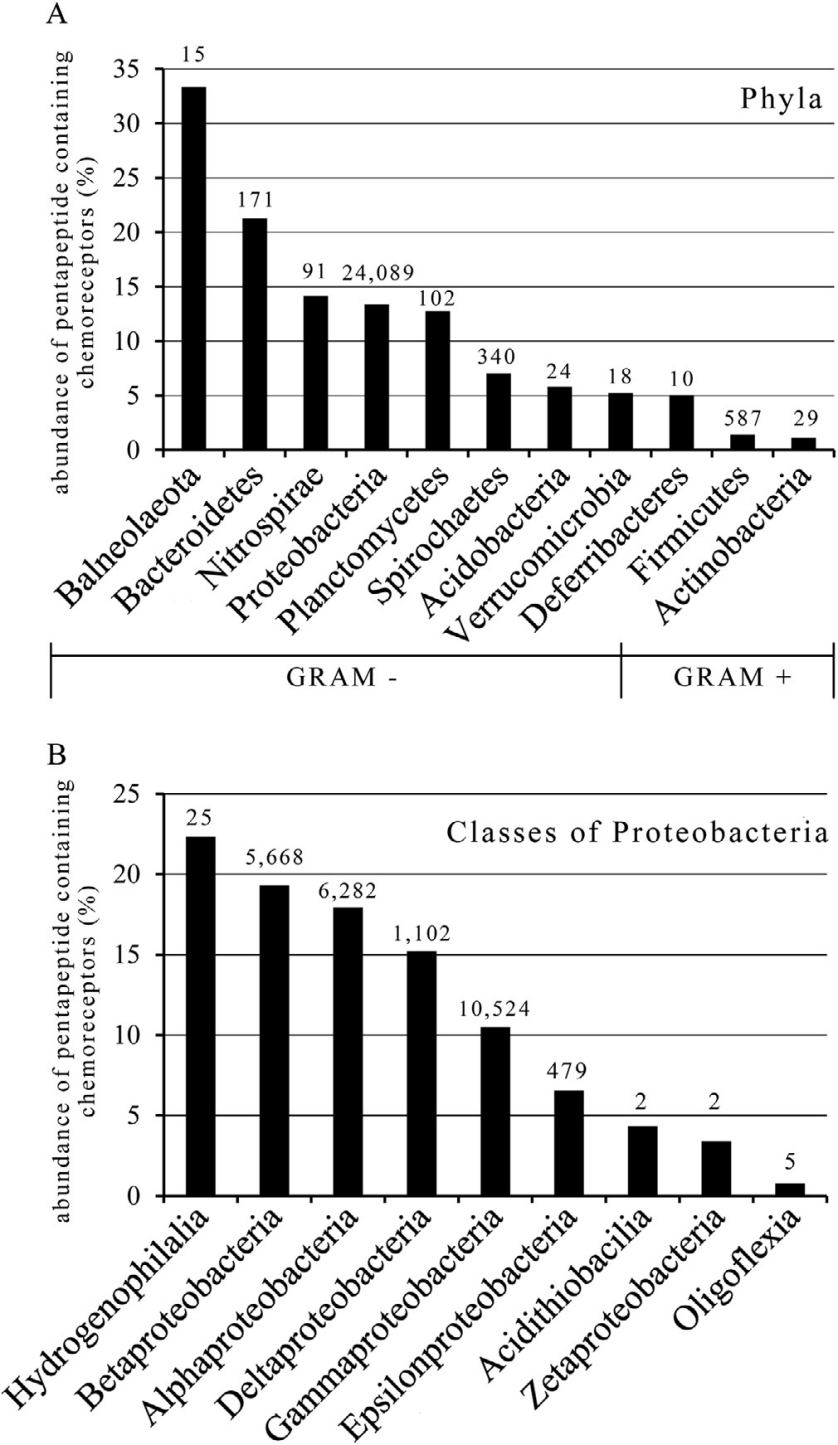
Abundance of pentapeptide containing chemoreceptors with respect to the total number of chemoreceptors in different phyla (A) and classes of the phylum Proteobacteria (B). The total number of pentapeptide containing chemoreceptors is indicated above each column.

**Fig. 7:**
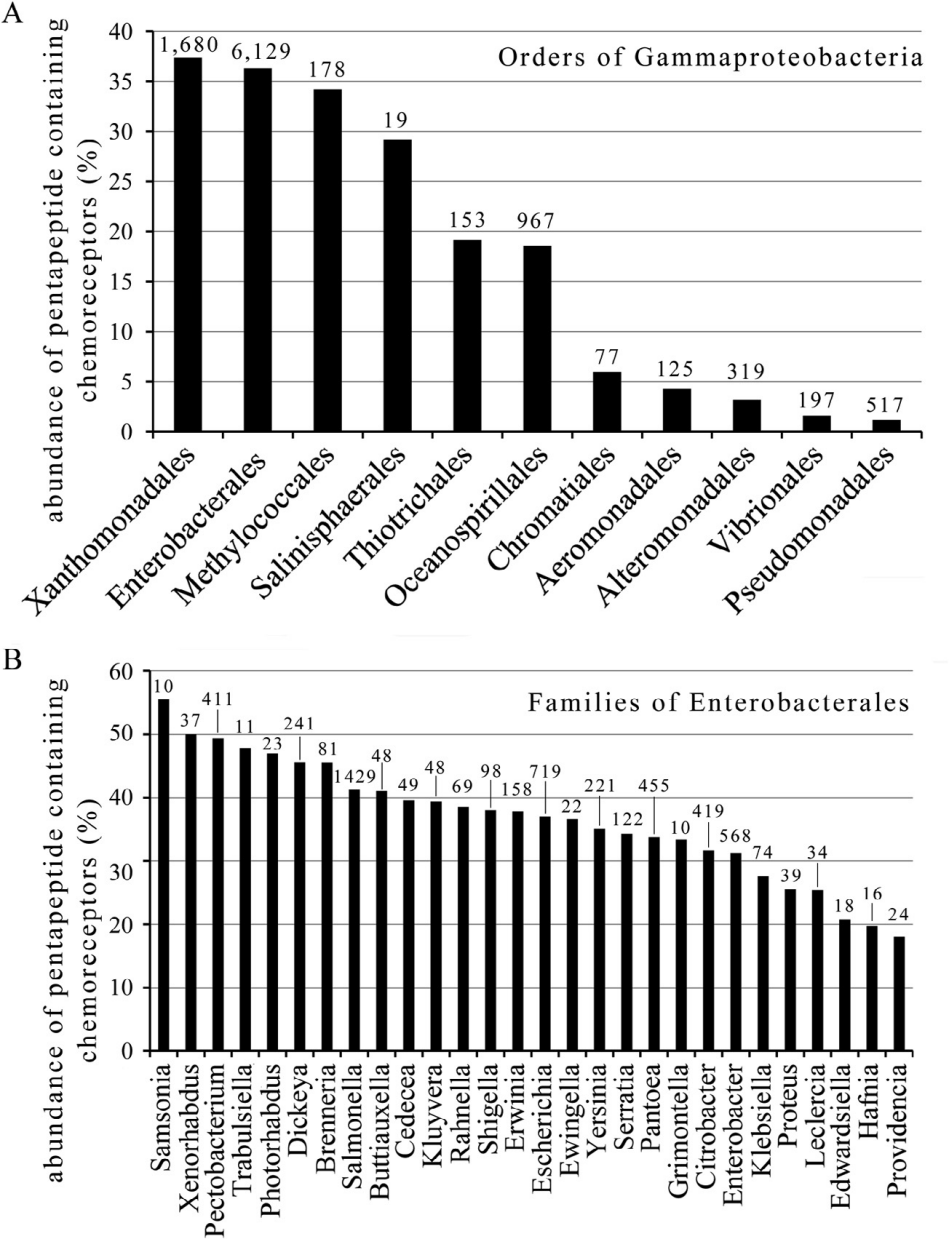
Abundance of pentapeptide containing chemoreceptors with respect to the total number of chemoreceptors in different orders of Gammaproteobacteria (A) and families of Enterobacterales (B). The total number of pentapeptide containing chemoreceptors is indicated above each column.

Pentapeptide containing chemoreceptors have been detected in 11 different phyla (Fig. 6A) extending thus the initial observation that such receptors are found in Proteobacteria and Spirochaetes [9]. Of note is the high abundance in Balneolaeota and Bacteroidetes. Both phyla are very close and the separation of the new phylum Balneolaeota from Bacteroidetes has been proposed recently [42]. Interestingly, Bacteriodetes are among the major components of the human microbiota, especially in the gastrointestinal tract [43], suggesting a link between lifestyle and the abundance of pentapeptides. The 9 phyla of Gram-negative bacteria showed an abundance of pentapeptide containing chemoreceptors between 5 and 33 %. However, this value was with approximately 1% significantly lower in the two phyla of Gram-positive bacteria, Firmicutes and Actinobacteria (Fig. 6A). Proteobacteria was the most populated phylum with more than 24,000 pentapeptide containing chemoreceptors, corresponding to an abundance of 13.4%. All nine classes of Proteobacteria were found to harbour pentapeptides and the abundance in Gammaproteobacteria is comparable to that of the bacterial average (Fig. 6B). Xanthomonadales and Enterobacterales were identified as the two orders of Gammaproteobacteria that showed an abundance of pentapeptide superior to 35% (Fig. 7A). In fact, almost a quarter of all pentapeptide containing chemoreceptors are present in Enterobacterales, providing further support for the notion that pentapeptide containing chemoreceptors are abundant in species that inhabit the intestine. In contrast, the abundance of pentapeptide containing chemoreceptors in Pseudomonadales, that are to a large degree free living environmental bacteria, is with approximately 1% very low (Fig. 7A). Fig. 7B shows that the major families of the order Enterobacterales are all characterized by an elevated percentage of pentapeptide containing chemoreceptors. Of note are the Pectobacterium and Dickeya families, constituted by mainly plant pathogenic strains, for which almost every second chemoreceptor contains a pentapeptide.

Table 1 shows the abundance of pentapeptide containing chemoreceptors in populated orders (sum of chemoreceptors of the individual strains is superior to 400). An absence of pentapeptide containing chemoreceptors was noted in Halobacteriales and a low frequency was observed in Bacillales (0.19%), Vibrionales (1.62%) and Clostridiales (2.89%). The non-proteobacterial orders with the highest abundance were the Spirochaetes Brachyspiraceae (9.34%) and Leptospiraceae (7.27%).

**Table 1:** Abundance of pentapeptide containing chemoreceptors in different well-populated orders.

Order	Abundance of pentapeptide containing receptors (%)	Total chemoreceptors of the Order	Class	Phylum
Caulobacterales	38.22	1316	Alphaproteobacteria	Proteobacteria
Xanthomonadales	37.41	4485	Gammaproteobacteria	Proteobacteria
Enterobacterales	36.36	16,839	Gammaproteobacteria	Proteobacteria
Methylococcales	34.24	517	Gammaproteobacteria	Proteobacteria
Nitrosomonadales	25.03	947	Betaproteobacteria	Proteobacteria
Burkholderiales	21.27	24,364	Betaproteobacteria	Proteobacteria
Rhizobiales	21.37	19,706	Alphaproteobacteria	Proteobacteria
Desulfuromonadales	20.96	3069	Deltaproteobacteria	Proteobacteria
Sphingomonadales	20.23	3069	Alphaproteobacteria	Proteobacteria
Rhodobacterales	19.57	3409	Alphaproteobacteria	Proteobacteria
Thiotrichales	19.14	794	Gammaproteobacteria	Proteobacteria
Oceanospirillales	18.58	5199	Gammaproteobacteria	Proteobacteria
Desulfobacterales	17.66	1467	Deltaproteobacteria	Proteobacteria
Desulfovibrionales	13.64	1972	Deltaproteobacteria	Proteobacteria
Brachyspiraceae	9.34	503	Spirochaetales	Spirochaetes
Leptospiraceae	7.27	2091	Spirochaetales	Spirochaetes
Rhodocyclales	7.13	1249	Betaproteobacteria	Proteobacteria
Campylobacterales	6.6	7208	Epsilonproteobacteria	Proteobacteria
Myxococcales	6.38	1301	Deltaproteobacteria	Proteobacteria
Chromatiales	5.96	1276	Gammaproteobacteria	Proteobacteria
Spirochaetaceae	4.53	1413	Spirochaetes	Spirochaetes
Aeromonadales	4.34	2883	Gammaproteobacteria	Proteobacteria
Neisseriales	4.3	2048	Betaproteobacteria	Proteobacteria
Alteromonadales	3.16	10,094	Gammaproteobacteria	Proteobacteria
Rhodospirillales	3.14	6314	Alphaproteobacteria	Proteobacteria
Clostridiales	2.89	15,562	Clostridia	Firmicutes
Halanaerobiales	2.53	475	Clostridia	Firmicutes
Selenomonadales	1.72	1516	Negativicutes	Firmicutes
Vibrionales	1.62	11,825	Gammaproteobacteria	Proteobacteria
Pseudomonadales	1.17	44,040	Gammaproteobacteria	Proteobacteria
Thermoanaerobacterales	1.13	533	Clostridia	Firmicutes
Lactobacillales	0.93	643	Bacilli	Firmicutes
Micromonosporaceae	0.82	612	Actinobacteria	Actinobacteria
Oscillatoriales	0.5	402	Cyanophyceae	Cyanobacteria
Geodermatophilaceae	0.19	521	Actinobacteria	Actinobacteria
Bacillales	0.19	22,042	Bacilli	Firmicutes
Halobacteriales	0	2730	Halobacteria	Euryarchaeota

## 9 Predominant negative net charge of the pentapeptide

**Fig. 8:**
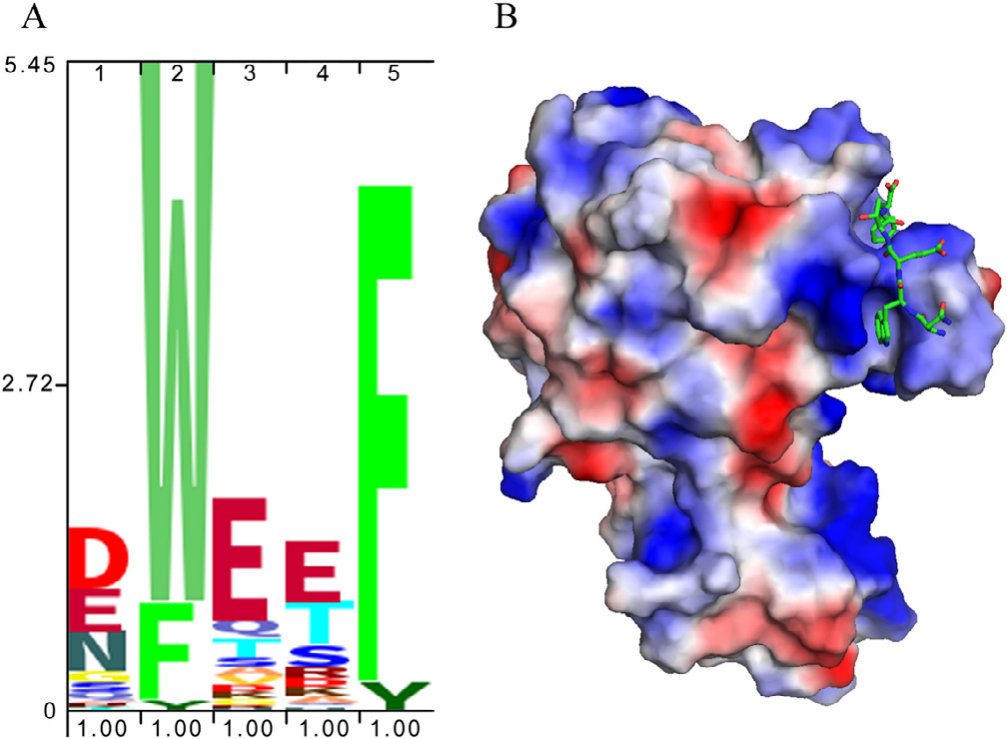
The consensus motif of pentapeptides identified in this study (A) and surface charge representation of the 3D structure of *S. typhimurium* CheR in complex with the NWETF pentapeptide (B). PDB ID: 1BC5, Red: negative charge, blue: positive charge. (For interpretation of the references to colour in this figure legend, the reader is referred to the web version of this article.)

The consensus motif of all pentapeptides identified is shown in Fig. 8A. Tryptophan and phenylalanine are the predominant residues in positions 2 and 5, respectively, a finding that has already been made in the initial study [9]. However, negatively charged amino acids are primarily found at the remaining positions. The dominance of negatively charged amino acids was less obvious in the initially reported consensus [9]. The structure of the *S. typhimurium* CheR [17] (Fig. 8B) shows that the NWETF pentapeptide binding site has a positive surface charge and charge attraction between a negatively charged pentapeptide and a positively charged binding site at CheR may contribute to binding.

## 10 Conclusions

Data show that the phylogenetic distribution of pentapeptide containing chemoreceptors is wider than initially thought and involve 11 phyla. Multiple and interconnected lines of evidence show that pentapeptide chemoreceptors are particularly abundant in Gram-negative bacteria that inhabit the intestine. Firstly, phylogenetic analyses show a high percentage of pentapeptide containing chemoreceptors in Bacteroidetes and Enterobacterales, two taxonomic categories that are composed by many strains that inhibit the intestine. Secondly, the classification of strains according to lifestyle has revealed a high abundance of pentapeptides in strains that inhibit the human intestine. Thirdly, there is a very low abundance of pentapeptide containing chemoreceptors in Pseudomonads that are to a large degree free living environmental bacteria. Experimental research is now required to assess the potential role of pentapeptide containing chemoreceptors in host interaction.

## 11 Materials and methods

Retrieval of chemoreceptor sequences and identification of chemoreceptors that contain a C-terminal pentapeptide: Chemoreceptors are defined as proteins that contain a MCPsignal domain (Pfam PF00015) [44]. Chemoreceptors were retrieved from the TrEMBL section of UniProt Knowledgebase (UniProtKB, release 2019_11) [45]. At the time of sequence retrieval there were 179,812,129 protein sequences that belonged to 140,835 proteomes. Subsequently, chemoreceptors were retrieved that matched the C-terminal sequence xZxxZ, where x corresponds to any residue and Z to F, W or Y. This criterion was based on the initial study by Perez and Stock [9]. Since the random probability of this motif at the C-terminus of proteins is minor, no constraints were put on the linker sequence length. The sequences of pentapeptide containing chemoreceptors formed the 5Pset dataset.

Classification of chemoreceptors according to cellular compartment and type of LBD: Proteins that contained at least one transmembrane region, as annotated in the UniProt Topology section, were classified as transmembrane proteins whereas the remaining receptors formed the group of soluble chemoreceptors. The complete set of chemoreceptors as well as members of the 5Pset were analysed for the presence of at least one of the following LBDs: TarH (PF02203), CHASE3 (PF05227), 4HB_MCP_1 (PF12729), PAS_9 (PF13426), PAS_4 (PF08448), PAS_3 (PF08447), sCACHE_2 (PF17200), sCACHE_3_3 (PF17202), protoglobin (PF11563), CZB (PF13682), GAF (PF01590), Pilj (PF13675), dCACHE_2 (PF08269), dCACHE_3 (PF14827), HBM (PF16591), NIT (PF08376), Cache_3-Cache_2 (PF17201) and dCache_1 (PF02743). These domains have been identified as the most abundant LBDs in chemoreceptors [35].

Taxonomic classification of chemoreceptors: The taxonomic category of chemoreceptor containing strains was extracted from Names and Taxonomy section of UniProt. A Python dictionary was created adding the frequency of the pentapeptide containing sequences to each of the taxonomic categories. The abundance of pentapeptide containing chemoreceptors with respect to the total number of chemoreceptors was calculated.

Generation of a pentapeptide motif consensus sequence: The pentapeptide motif sequence was extracted from each of the sequences from the 5Pset entries and a fasta file was created containing the pentapeptide sequence list. This file was used for multiple sequence alignment by MUSCLE [46], the generation of an HMM with HMMbuild (http://www.hmmer.org) and the generation of a Sequence Logo with Skylign [47]*.*

Identification of the bacterial habitat: Bacterial strains that contain C-terminal pentapeptides were submitted to an analysis by the fusionDB database [41] that assigns the preferred habitat and classifies strains into habitat categories. For each of the categories the abundance of pentapeptide containing chemoreceptors with respect to their total number was calculated. These operations were done based on in-house developed Python scripts.

Display of three dimensional structures: Images of protein structures were produced by the PyMOL Molecular Graphics System, Version 2.2.0 Schrödinger, LLC.

## CRediT authorship contribution statement

**Álvaro Ortega:** Methodology, Software, Data curation. **Tino Krell:** Conceptualization, Writing - review & editing.

## Declaration of Competing Interest

The authors declare that they have no known competing financial interests or personal relationships that could have appeared to influence the work reported in this paper.

## Footnotes

1 Abbreviations: LBD: ligand binding domain
