# Supplementary material for: Chemoreceptors with C-terminal pentapeptides for CheR and CheB binding are abundant in bacteria that maintain host interactions
Source: Comput Struct Biotechnol J. 2020 Jul 16;18:1947–55. doi: 10.1016/j.csbj.2020.07.006 (PMC7390727; doi:10.1016/j.csbj.2020.07.006)

Supplementary Figures

to

**Chemoreceptors with C-terminal pentapeptides for CheR and CheB binding are**

**abundant in bacteria that maintain host interactions**

by

Alvaro Ortega and Tino Krell

**Supp. Fig. 1) Archaeal chemoreceptors with pentapeptides.** Shown are consensus secondary structure predictions of the C-terminal part of the chemoreceptors using the NPSA algorithm [1] (e: β-strand, h: α-helix, C: coil). The terminal pentapeptide is shown in green. For chemoreceptors 1 to 3 the pentapeptide is fused to the MCPsignal domain via an unstructured linker. In addition, the non-aromatic residues of the pentapeptide of chemoreceptors 1 to 3 are mostly negatively charged, corresponding to the consensus sequence identified (see Fig. 8A). In contrast, the pentapeptide is fused directly to the methylaccepting domain of chemoreceptor 4 and does not contain negatively charged amino acids. In addition, BLAST searches using these four chemoreceptors did not identify bacterial homologues indicating that these receptors are not the result of an unwanted bacterial contamination.

1. **Chemoreceptor A0A497H153**

Thermoplasmata archaeon

Sequence: AKIIKTIDEVAFQTNLLALNAAVEAARAGESGKGFAVVAEEVRNLAMRSAEAAKGTAALIEGAQANAEQGVRVSAEVAKIFAQIKTSIDNTTHLIAEVSTATEEQAQGIYEINQAVSQVDTVTQANAAHSEEMTSVSVELAAEAESLQNMVNTLLGIVEGRRTDSDDRYRSYPAIEDRSAQERDRRGLLDEPEEFSATAKPKDCDEEFDDF

10 20 30 40 50 60

| | | | | |

UNK_1002170 AKIIKTIDEVAFQTNLLALNAAVEAARAGESGKGFAVVAEEVRNLAMRSAEAAKGTAALI

DSC cchhhhhhhhhhhhhhhhhhhhhhhhhhhhhccchhhhhhhhhhhhhhhhhhhhhhhhhh

MLRC cceeehhhhhhhhhhhhhhhhhhhhhhhccccccehehhhhhhhhhhhhhhhhhhhhhhh

PHD cccehhhhhhhhhhhhhhhhhhhhhhhhcccccceeehhhhhhhhhhhhhhhhhhhhhhh

70 80 90 100 110 120

| | | | | |

UNK_1002170 EGAQANAEQGVRVSAEVAKIFAQIKTSIDNTTHLIAEVSTATEEQAQGIYEINQAVSQVD

DSC hhhhcchhhhhhhhhhhhhhhhhhhhhhhhhhhhhhhhhhhhhhhhhhhhhhhhhhhhhh

MLRC hhccccccchhhhhhhhhhhhhhhhhhhhhhhhhhhhhhhhhhhhhhhhhhhhhhhhhhh

PHD hhhhhhhcccceeehhhhhhhhhhhhhhhhhhhhhhhhhhhhhhhhhhhhhhhhhhhhhh

130 140 150 160 170 180

| | | | | |

UNK_1002170 TVTQANAAHSEEMTSVSVELAAEAESLQNMVNTLLGIVEGRRTDSDDRYRSYPAIEDRSA

DSC hhhhhhhhhhhhhhhhhhhhhhhhhhhhhhhhhhhhhhcccccccccccccccchhhhhh

MLRC hhhhhcccchhhhhhhhhhhhhhhhhhhhhhhhhhhhhccccccccccccccccccccch

PHD hhhhhccchhhccchhhhhhhhhhhhhhhhhhhhhhhhhcccchhhccccccccccccch

190 200 210

| | |

UNK_1002170 QERDRRGLLDEPEEFSATAKPKDCDEEFDDF

DSC hhhhhhhhccchhhhhccccccccccccccc

MLRC hhhhccccccccccccccccccccchchccc

PHD hhhcccccccccccccccccccccchhhccc

1. **Chemoreceptor A0A522YRI4**

Candidatus Nitrosotenuis sp.

YAQTKIMVLLTAIIGVLSGIALAVIILRSVTKSIGEVMEGLTEGSDQVHTASGQLSETSQ

QMAEGASEQAASIEETSSSLEEISSMTKHNADNSNAVSNLMSETKRGVDKGSGQMKELVT

AMGDIKKASDDIAKIIKVIEEIAFQTNLLALNAAVEAARAGEHGKGFAVVAEEVRNLAQR

AGTASKDIAQLIQNAVSKADSGNEITVQVAKSLDDIATGVKKAGDLAAEVAAASVEQAQG

VEQINKAVTQMDSVTQANAANAEEAASASEELSAQAELLNGHVQQLAAVIFGGEHGTHAP

AASLAKKPAPKAASKPMGKPMVKPKGLPAPRKAVPAPASAPASKGPKSVKAEEVIPFDDD

DFKEF

10 20 30 40 50 60

| | | | | |

UNK_1040410 YAQTKIMVLLTAIIGVLSGIALAVIILRSVTKSIGEVMEGLTEGSDQVHTASGQLSETSQ

DSC cccchhhhhhhhhhhhhccccceeehhhhhhhhhhhhhhhhhcchhhhhhhcchhhhhhh

MLRC cchhhhhhhhhhhhhhhhhhhhhhhhhhhhhhhhhhhhhhhhccchhhhhcccchhhhhh

PHD cccceeeeeeehhhhhhhhhhhhhhhhhhhhhhhhhhhhhhhhchhhhhhhhhhhhhhhh

70 80 90 100 110 120

| | | | | |

UNK_1040410 QMAEGASEQAASIEETSSSLEEISSMTKHNADNSNAVSNLMSETKRGVDKGSGQMKELVT

DSC hhhhhhhhhhhhhhhhhhhhhhhhhhhhhhhhhhhhhhhhhhhhhhhhhhhhhhhhhhhh

MLRC hhhhhhhhhhhhhhhhhhhhhhhhhhhhhcccchhhhhhhhhhhhhhhhccchhhhhhhh

PHD hhhhhhhhhhhhhhhhhhhhhhhhhhhhhhhhhhhhhhhhhhhhhhhhhhchhhhhhhhh

130 140 150 160 170 180

| | | | | |

UNK_1040410 AMGDIKKASDDIAKIIKVIEEIAFQTNLLALNAAVEAARAGEHGKGFAVVAEEVRNLAQR

DSC hhhhhhhhhhhhhhhhhhhhhhhhhhhhhhhhhhhhhhhhhhhccceehhhhhhhhhhhh

MLRC hhhhhhhhhhhhhhhhhhhhhhhhhhhhhhhhhhhhhhhhcccccchhhhhhhhhhhhhh

PHD hhhhhhhchhhhhhhhhhhhhhhhhhhhhhhhhhhhhhhhcccccceeehhhhhhhhhhh

190 200 210 220 230 240

| | | | | |

UNK_1040410 AGTASKDIAQLIQNAVSKADSGNEITVQVAKSLDDIATGVKKAGDLAAEVAAASVEQAQG

DSC hhhhhhhhhhhhhhhhhhhhcchhhhhhhhhhhhhhhhhhhhhhhhhhhhhhhhhhhhhh

MLRC hhhhhhhhhhhhhhhhhcccccchhhhhhhhhhhhhhhhhhhhhhhhhhhhhhhhhhhhh

PHD hhhhhhhhhhhhhhhhhhhcccchhhhhhhhhhhhhhhhhhhhhhhhhhhhhhhhhhhhh

250 260 270 280 290 300

| | | | | |

UNK_1040410 VEQINKAVTQMDSVTQANAANAEEAASASEELSAQAELLNGHVQQLAAVIFGGEHGTHAP

DSC hhhhhhhhhhhhhhhhhhhhhhhhhhhhhhhhhhhhhhhhhhhhhhheeeeccccccccc

MLRC hhhhhhhhhhhhhhhhhhhhhhhhhhhhhhhhhhhhhhhhhhhhhhhhhheccccccccc

PHD hhhhhhhhhhhhhhhhhhhhchhhhhhhhhhhhhhhhhhhhhhhhhhhheecccccchhh

310 320 330 340 350 360

| | | | | |

UNK_1040410 AASLAKKPAPKAASKPMGKPMVKPKGLPAPRKAVPAPASAPASKGPKSVKAEEVIPFDDD

DSC hhhhhhccccchhccccccccccccccccccccccccccccccccccccccceecccccc

MLRC hhhhhcccccccccccccccccccccccccccccccccccccccccccccccceeecccc

PHD hhhhhccccccccccccccccccccccccccccccccccccccccccccceeeeeecccc

UNK_1040410 DFKEF

DSC ccccc

MLRC ccccc

PHD ccccc

1. **Chemoreceptor A0A524QNV2**

Methanothrix sp.

MFKNMKVGSKIIGGFAIVLVLLVVVAYVGYNGLSNVTDRVDKADNVNLLINSILATRQQE

KNFIIRGDKKYVDTVAEQVEALKKQANETRAKFNDPVNISQMDEVISSAGGYKKAFDDYV

KLSEQQKVADDNMVKAAREVNEVADAIRQEQKTQFEELTKAGATAAQIEDKLIKSDDANR

IIKWVLESRRQEKNFIIRSDRNYADLVNKHAEDIVNLAKDMKSRFNQAQNQQQADNIITA

TQAYKAAFNNYVIFKDKQVEADAEMVASARSVQEVCDKARADQKAKMDGQISMANSIALI

SSIIAIILGSGLAFVITRGITKPLNRAIADLSEGAGQVASASGQVSEASQSLAEGASEQA

ASIEETSSSLEEMSSMTKQNADNAGQANNLMKEVNHVVSDANSSMTELTTSMQEISRASD

ETQKVVKTIDEIAFKTNLLALNAAVEAARAGEAGAGFAVVAEEVRNLALRSADAAKNTAD

LIEGTVKKVKAGSELVAKTNNAFIKVAESSSKVGELIAEVTAASHEQAQGIEQTNTAVAE

MDKVTQQNAANAEESASASEELTAQAEQMMGTVGELTALVGGNSSKETVSRQQTLGGKNK

IGAKRTLQASAGKAKVQTVRKTKPAQISHRAEVRPDQVIPLDEDDFKGF

370 380 390 400 410 420

| | | | | |

UNK_1103080 ASIEETSSSLEEMSSMTKQNADNAGQANNLMKEVNHVVSDANSSMTELTTSMQEISRASD

DSC hhhhhhhhhhhhhhhhhhhhhhhhhhhhhhhhhhhhhhhhhhhhhhhhhhhhhhhhhhhh

MLRC hhhhhhhhhhhhhhhhhhhcccchhhhhhhhhhhhhhhhhhhhhhhhhhhhhhhhhhhhh

PHD hhhhhcchhhhhhhhhhhhhhhhhhhhhhhhhhhhhhhhhhhhhhhhhhhhhhhhhhhhh

430 440 450 460 470 480

| | | | | |

UNK_1103080 ETQKVVKTIDEIAFKTNLLALNAAVEAARAGEAGAGFAVVAEEVRNLALRSADAAKNTAD

DSC hhhhhhhhhhhhhhhhhhhhhhhhhhhhhhhhhccchhhhhhhhhhhhhhhhhhhhhhhh

MLRC hhhhhhhhhhhhhhhhhhhhhhhhhhhhhhccccccchehhhhhhhhhhhhhhhhhhhhh

PHD hhhhhhhhhhhhhhhhhhhhhhhhhhhhhhhhcccchhhhhhhhhhhhhhhhhhhhhhhh

490 500 510 520 530 540

| | | | | |

UNK_1103080 LIEGTVKKVKAGSELVAKTNNAFIKVAESSSKVGELIAEVTAASHEQAQGIEQTNTAVAE

DSC hhhhhhhhhhcchhhhhhhhhhhhhhhhhhhhhhhhhhhhhhhhhhhhhhhhhhhhhhhh

MLRC hhhhhhhhhcccceeehhchhhhhhhhhhhhhhhhhhhhhhhhhhhhhhchhhhhhhhhh

PHD hhhhhhhhhccccchhhhhhhhhhhhhhhhhhhhhhhhhhhhhhhhhhhchhhhhhhhhh

550 560 570 580 590 600

| | | | | |

UNK_1103080 MDKVTQQNAANAEESASASEELTAQAEQMMGTVGELTALVGGNSSKETVSRQQTLGGKNK

DSC hhhhhhhhhhhhhhhhhhhhhhhhhhhhhhhhhhhhhhhccccccchhhhhhhhcccccc

MLRC hhhhhhhhccchhhhhhhhhhhhhhhhhhhhhhhhhhhhhcccccccccchhcccccccc

PHD hhhhhhhchhhhhhhhhhhhhhhhhhhhhhhhhhhhhhhheccccccccccccccccccc

610 620 630 640

| | | |

UNK_1103080 IGAKRTLQASAGKAKVQTVRKTKPAQISHRAEVRPDQVIPLDEDDFKGF

DSC cchhhhhhhhhccceeehhhhccchhhhhhhcccccccccccccccccc

MLRC ccchhhhchcccccchhhhcccccccccccccccccceeeccccccccc

PHD ccccccccccccccccccccccccccccccccccccccccccccccccc

1. Chemoreceptor A0A2V2NDA1

Methanospirillum stamsii

MKNKITSWLDNATIGRKISVISLILVIVPALIVGFVAYSSAESAIKNDIQTNLEVQVDDI

NDESATVYDLTLVKVKSDLNVFREFFYEKGDAAIVNDKMVLGSSYVVNDNFQIVDEVQKL

LGGAATVFQKEGDQAIRISTNVIGEDGKRAIGTSVSDKVYDEVINKGQTYYGTATVVGKE

YITAYEPIKDKSGNIIGILFVGVEEDSTIGLLEDQIKAKKIGQDGYMYVLNSQGITVIHP

TNEGRNDSDLPFIKDIIAKKDGYLAYNYNGVEKVAAFSYFEPFDWIIVASSELDDFTGPL

DTIRNAIIIVIIIGVIAGIIVSYLFGRSISRRMDDLVRLAHMVKDGDLSGNITLSESNDE

IGVLGRAFAELVTTFRLFRDEVRTLSLAASSGNLNVRGDVSKFQGDYAVIIDGVNETVDA

MVTPLQAAMELCNSYAKGDFKARVNPDLHLEGDFIKFRDALNTIGTDISEALIAVSAQVE

EVSSKVDEVSSKVDEVTSETCEAYKSIEDVSEGTGQVARIAAAVNDLADKSGMNTQQILA

AMNDLSTTVSSVATKMEHVSVLTNNASELSENGMNAAGQAETGMKGIMQASSAIDQMNRE

ISEQMQEIGRIVDIISSIAEETNLLALNAAIEAARAGDAGLGFAVVAAEVKELANESQKS

AENIAGIISALQKKSAAMADAVSNSLSEVETGNVSVSKTLEIFNEIVTSISVIHANMGEV

AAASEEQAASVEEVTATVNEFSDMVNQTAKESIGLAAASEQSSAAVGQITTMVSQVNDSM

EYIHRIAGEAHESVQRIHEKMNRFKYY

610 620 630 640 650 660

| | | | | |

UNK_1132650 ISEQMQEIGRIVDIISSIAEETNLLALNAAIEAARAGDAGLGFAVVAAEVKELANESQKS

DSC hhhhhhhhhhhhhhhhhhhhhhhhhhhhhhhhhhhhhhhccceeeehhhhhhhhhhhhhh

MLRC hhhhhhhhhhhhhhhhhhhhhhhhhhhhhhhhhhhhcccccchhhhhhhhhhhhhhhhhh

PHD hhhhhhhhhhhhhhhhhhcchhhhhhhhhhhhhhhhhhccccceeehhhhhhhhhhhhhh

670 680 690 700 710 720

| | | | | |

UNK_1132650 AENIAGIISALQKKSAAMADAVSNSLSEVETGNVSVSKTLEIFNEIVTSISVIHANMGEV

DSC hhhhhhhhhhhhhhhhhhhhhhhhhhhhhhcchhhhhhhhhhhhhhhhhhhhhhhhhhhh

MLRC hhhhhhhhhhhhhhhhhhhhhhhhhhhhhcccchhhhhhhhhhhhhhhhhhhhhhhhhhh

PHD hhhhhhhhhhhhhhhhhhhhhhhhhhhhhhccchhhhhhhhhhhhhhhhhhhhhhhhhhh

730 740 750 760 770 780

| | | | | |

UNK_1132650 AAASEEQAASVEEVTATVNEFSDMVNQTAKESIGLAAASEQSSAAVGQITTMVSQVNDSM

DSC hhhhhhhhhhhhhhhhhhhhhhhhhhhhhhhhhhhhhhhhhhhhhhhhhhhhhhhhhhhh

MLRC hhhhhhhhhhhhhhhhhhhhhhhhhhhhhhhhhhhhhhhhhhhhhhhhhhhhhhhhhhhh

PHD hhhhhhhhhhhhhhhhhhhhhhhhhhhhhhhhhhhhhhhhhhhhhhhhhhhhhhhhhhhh

790 800

| |

UNK_1132650 EYIHRIAGEAHESVQRIHEKMNRFKYY

DSC hhhhhhhhhhhhhhhhhhhhhhhcccc

MLRC hhhhhhhhhhhhhhhhhhhhhhhhccc

PHD hhhhhhhhhhhhhhhhhhhhhhhcccc

1. Combet, C.; Blanchet, C.; Geourjon, C.; Deleage, G., NPS@: network protein sequence analysis. *Trends Biochem Sci* **2000,** 25, (3), 147-50.

**Supp. Fig. 2) The principal domains that are present at the C-terminal extension of the chemoreceptor signaling domain (Pf00015).** Data taken from Pfam [1].

1. **CZB domain (Pf13682)**


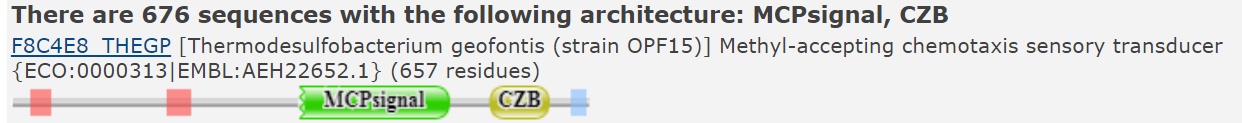


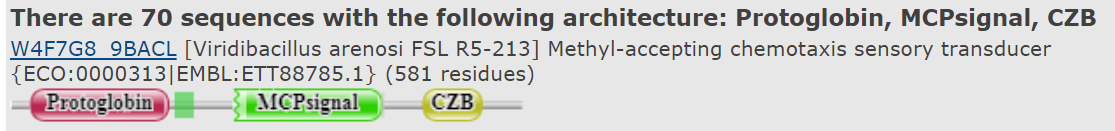


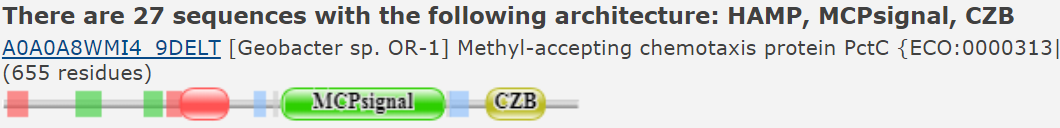


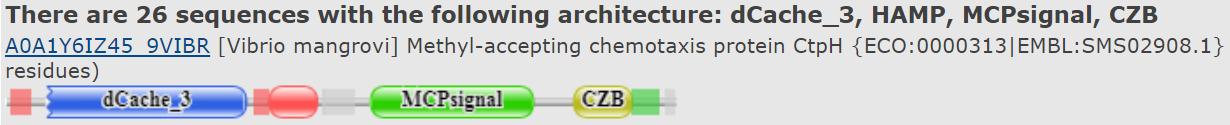


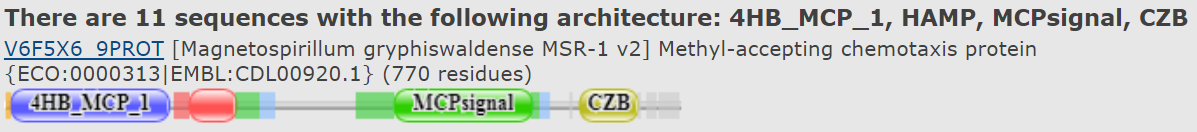


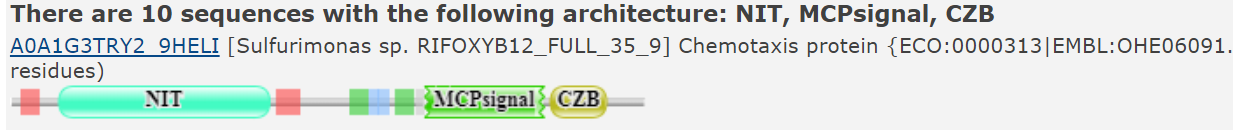


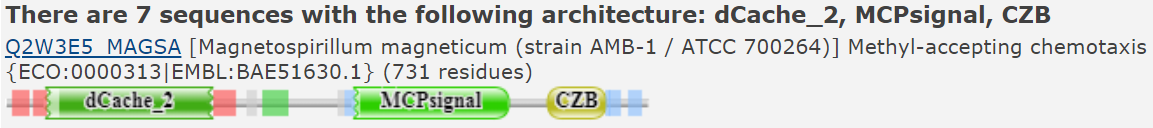


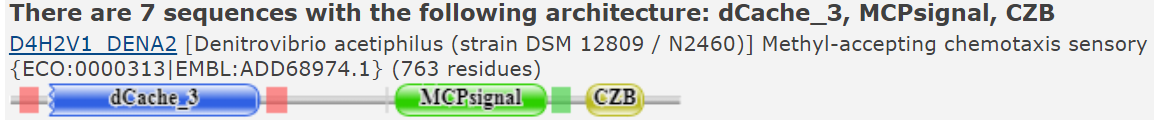


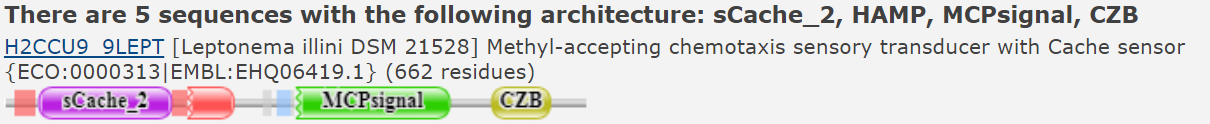


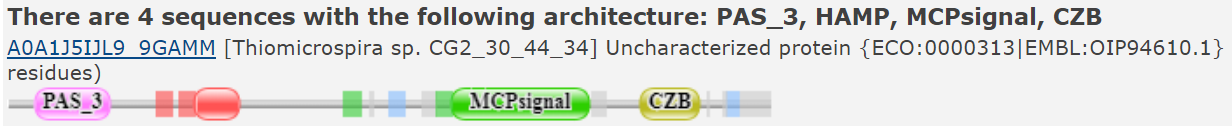


1. **PilZ domain (Pf07238)**


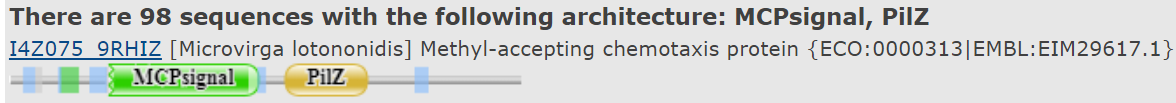


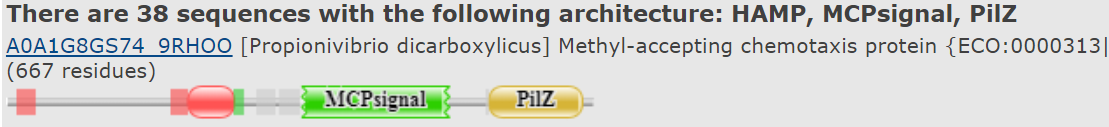


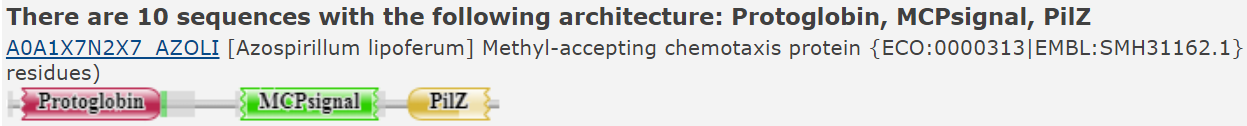


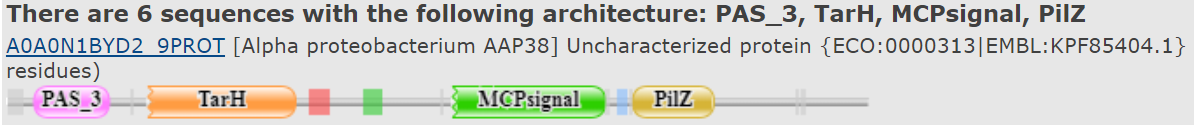


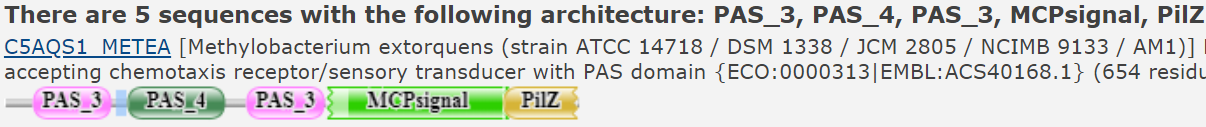


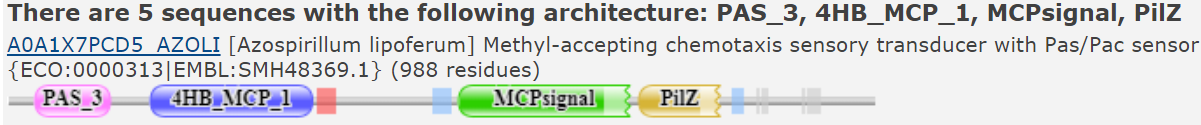


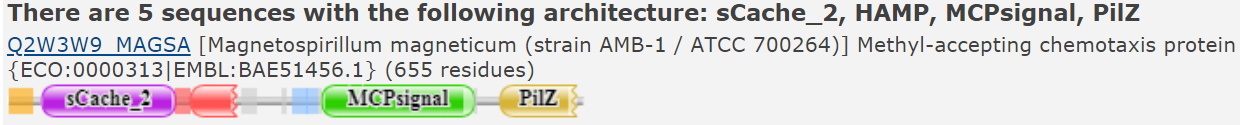


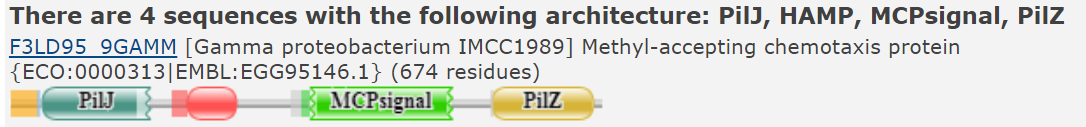


1. **Hemerythrin HHE cation binding domain (Pf01814)**


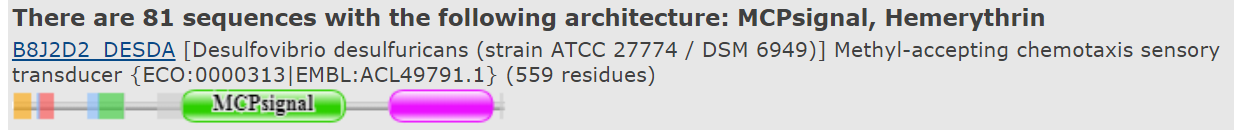


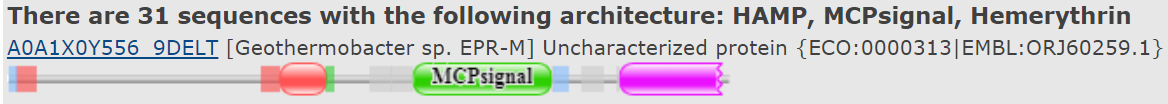


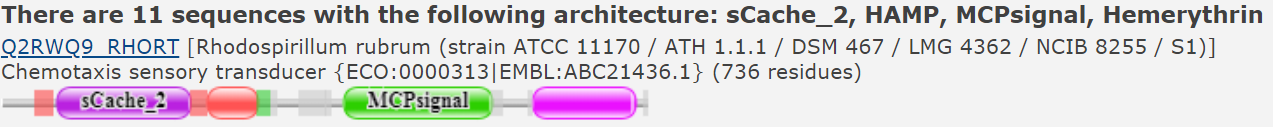


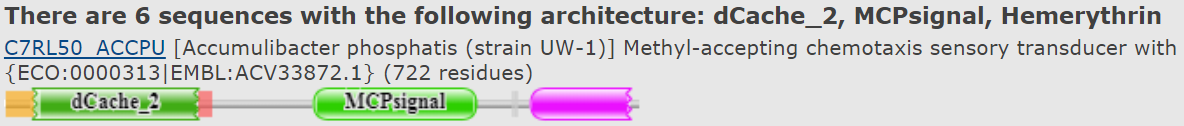


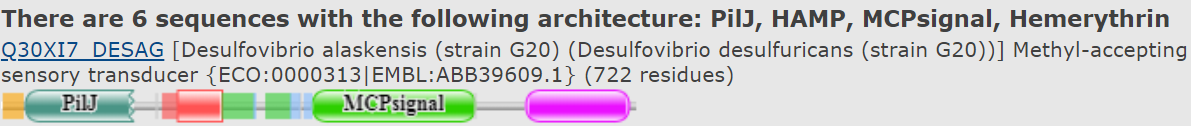


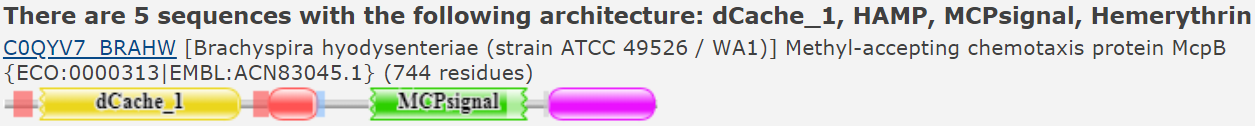


1. **sCACHE2 (Pf17200)**


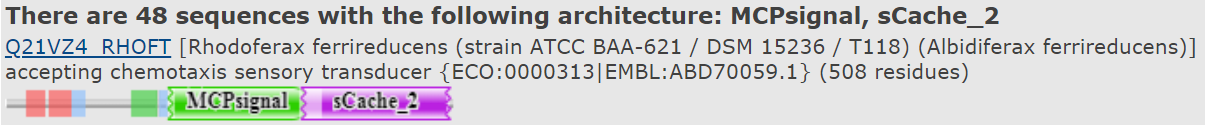


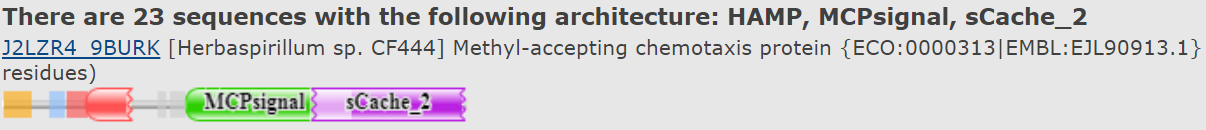


1. **Solute binding protein 5 (Pf00496)**


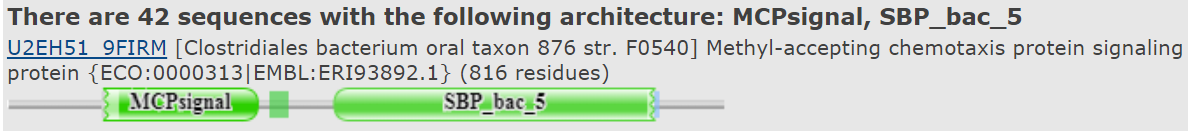


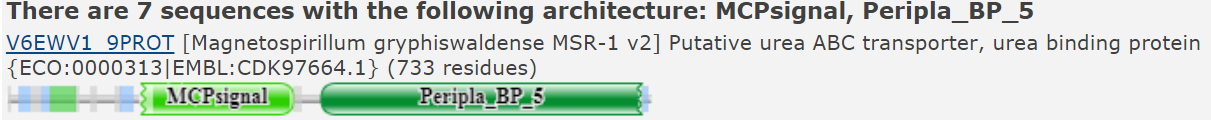


1. **Phosphonate binding protein (Pf12974)**


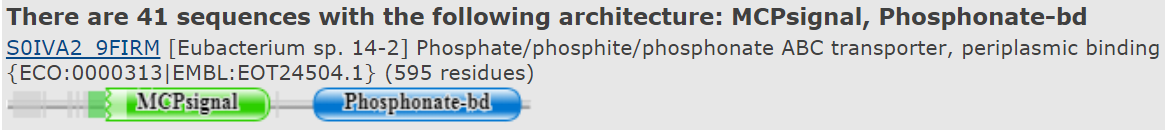


1. **Solute binding protein 3(Pf00497)**


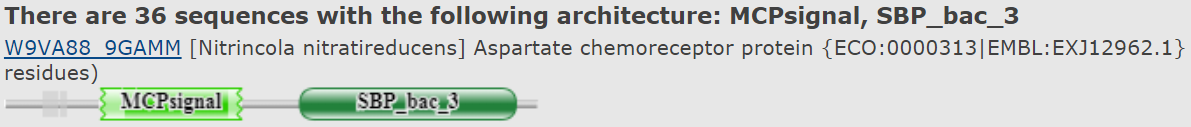


1. **Solute binding protein 4 (Pf13407)**


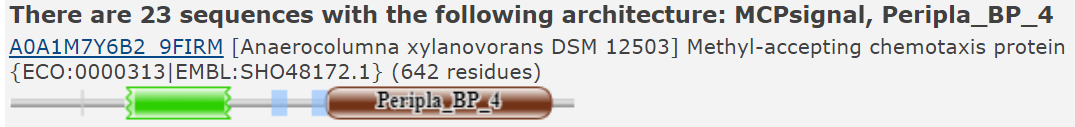


1. **Solute binding protein 8 (Pf13416)**


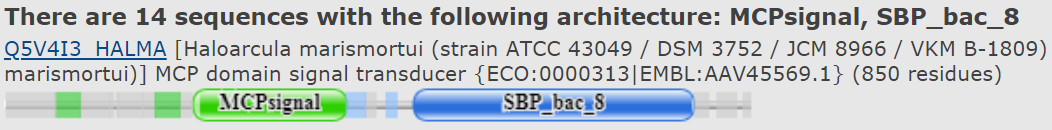


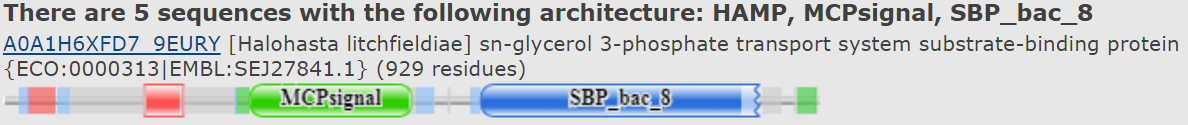


1. **Substrate binding domain of ABC-type glycine betaine transport system (Pf04069)**


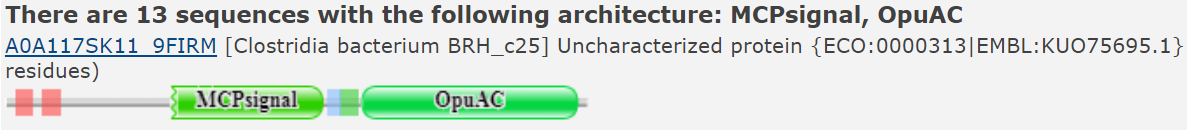


1. **PAS_3 (Pf08447)**


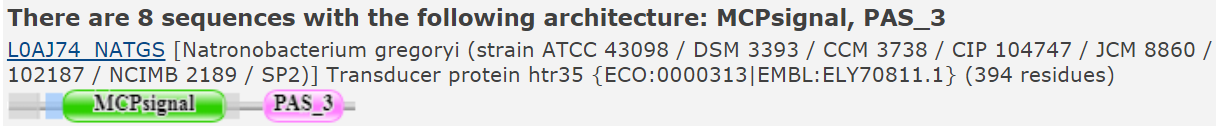


1. **Cache_3-Cache_2 (Pf17201)**


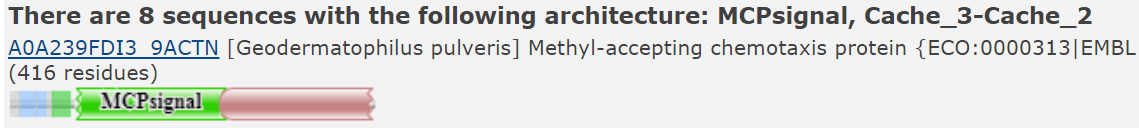


1. **HNOB Haem-NO-binding protein (Pf07700)**


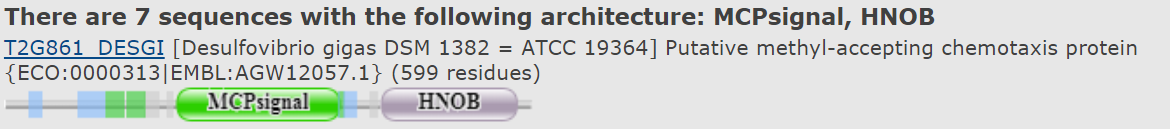


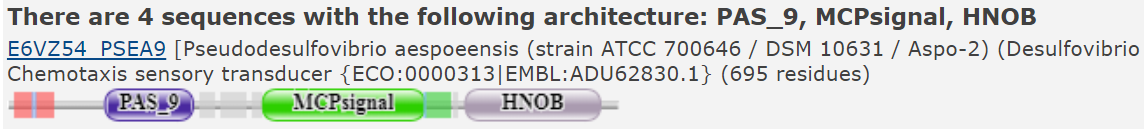


1. **FIST (Pf08495) and FIST_C (Pf10442)**


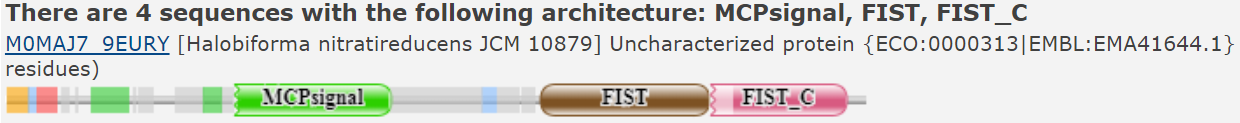


1. **Bacterial extracellular solute-binding protein, family 7 (Pf03480)**


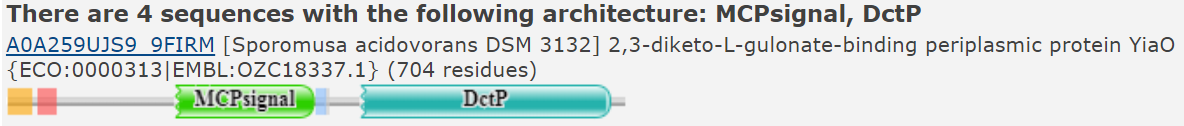


1. **Single Cache 3 (Pf17202)**


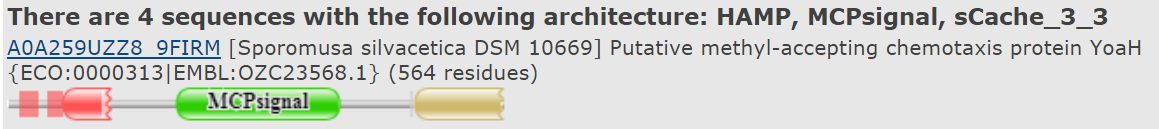


1. **4HB_MCP_1 four helix bundle domain (Pf12729)**


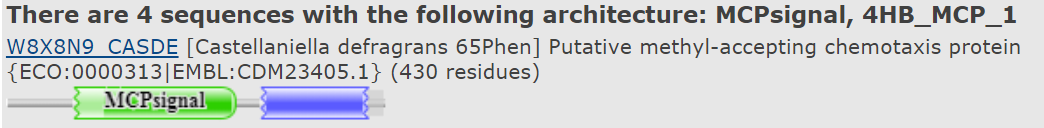

Supplement: Supplementary data 1 [file mmc1.docx]
